# The Structural Design of a New Graftable Antioxidant and the Theoretical Study of Its Role in the Cross-Linking Reaction Process of Polyethylene

**DOI:** 10.3390/polym17040546

**Published:** 2025-02-19

**Authors:** Yang Du, Hui Zhang, Chi Deng, Xia Du, Yan Shang, Xuan Wang, Qingguo Chen, Zesheng Li

**Affiliations:** 1 Key Laboratory of Engineering Dielectrics and Its Application of Ministry of Education & School of Material Science and Chemical Engineering, Harbin University of Science and Technology, Harbin 150080, China; duyang950711@163.com (Y.D.); dengchi@potevio.com (C.D.); duxia62@126.com (X.D.); shangyan1972@126.com (Y.S.); qgchen@hrbust.edu.cn (Q.C.); 2 Key Laboratory of Cluster Science of Ministry of Education & School of Chemistry, Beijing Institute of Technology, Beijing 100081, China; zeshengli@bit.edu.cn

**Keywords:** power cable, cross-linked polyethylene, antioxidant, grafting reaction, anti-oxidation mechanism

## Abstract

Cross-linked polyethylene (XLPE) insulation is used in most advanced power cable technology. However, in traditional cross-linking, the conductivity of the cross-linking system sharply increases due to the presence of additives (antioxidants and cross-linked agents). Therefore, reducing the number of antioxidants to further reduce conductivity is a very promising method. The structural design of a new dual-functional antioxidant 5-allyloxy-2-hydroxyl-3-tert-butylbenzophenone (5ATB) has been established. The antioxidant behavior and grafting reaction of 5ATB after photocatalysis under ultraviolet (UV) conditions were further studied using density functional theory (DFT). The reaction potential energy information of the six reaction channels at the B3LYP/6-311+G(*d,p*) level were obtained. The calculation results indicated that the reaction Gibbs energy barrier of 5ATB with O_2_ is approximately 0.48 eV lower than that of the polyethylene chain with O_2_ to achieve an anti-oxidative effect. Furthermore, the reaction-active site of 5ATB accepting H is located on the C of CH_2_ in a C=C double bond, as demonstrated by an analysis of NBO charge populations. The proposed mechanism has the potential to further expand the design concept of insulation materials for advanced future power cables.

## 1. Introduction

In order to reduce carbon emissions, there is a necessity to transition from the use of fossil fuels to renewable energy sources, which are considered to be cleaner. However, wind and solar power are typically most abundant in unpopulated areas that are distant from the end user. Reducing power loss during transportation over hundreds to thousands of kilometers is a decisive factor. The necessity for transmission lines arises from the intermittent nature of renewable energy, which necessitates the ability to reverse the direction of energy flow. It is evident that high-voltage direct current (HVDC) transmission lines fulfill the aforementioned requirements and thus represent a pivotal component of future power grids, which are designed to facilitate the integration of renewable energy sources in a seamless manner [1]. In the event of the transmission lines traversing densely populated areas or large bodies of water, it is imperative that they be submerged or buried underground. Therefore, an insulation layer must be employed to encase the cables. The insulation layer must be of a particularly high quality in order to withstand the high transmission voltage of hundreds of kilovolts. The research endeavors in this field have been focused on the augmentation of the transmission voltage to beyond 640 kV, a feat that is presently attainable [2].

Cross-linked polyethylene (XLPE) has become a staple in the field of advanced HVDC cable insulation due to its ability to meet the stringent requirements of very low conductivity. This product is derived from raw materials characterized by exceptional physical and chemical cleanliness, ensuring its reliability and performance in critical applications. The present study demonstrates the necessity of a degassing procedure to eliminate the byproducts of dicumyl peroxide (DCP) cross-linking [3,4]. With the potential for trace residues to augment the electrical conductivity of XLPE, the degassing procedure is imperative for ensuring the quality and reliability of the final product [5,6,7]. The utilization of ultraviolet (UV) radiation technology devoid of the byproducts of DCP cross-linking has the potential to serve as an effective solution for the fabrication of HVDC cable insulation. This assertion is substantiated by the successful implementation of this technology in the production of low-voltage cable insulation in China [8]. A comparison of the theoretical results obtained from the UV radiation cross-linking process with those of the conventional DCP cross-linking process reveals that the reaction energy barrier of H-abstraction on the polyethylene chain by benzophenone (UV radiation cross-linking) is 0.17 eV, which is 0.08 eV lower than that by cumyl peroxide radicals (DCP cross-linking) [9,10]. This finding suggests that UV radiation cross-linking technology may offer a distinct advantage over conventional DCP cross-linking technology in XLPE production.

Polyethylene is subject to oxidation during the production, storage, and utilization phases, resulting in a decline in its mechanical, physicochemical, and electrical properties. This deterioration contributes to a reduction in the operational stability and in-service life of the cable [11,12]. Antioxidants have been shown to retard the process of oxidation, thereby prolonging the lifespan of XLPE cable insulation [13]. To further reduce conductivity, the most common strategy for reducing the antioxidant content has been proposed. During the production process, oxidation can lead to the formation of radicals in polyethylene molecular chains and increase the cross-linking density, and the migration of antioxidants can reduce service life during the application process. The grafting of antioxidants with low molecular weights onto polyethylene was modified through the application of chemical methods with the objective of averting migration. This process was undertaken to improve the thermal oxidation and aging properties of the material [13,14,15,16]. It was found from Li and co-workers that compared with the industrial-produced voltage stabilizer UV-531 (4OB), the voltage stabilizer 4-allyloxy-2-hydroxylbenzophene (4AB) had a better effect on the electrical properties of XLPE [14]. Previous studies have also demonstrated that structures with a 2-hydroxy-benzophenone configuration can scavenge free radicals in the system, serving as antioxidants [15]. Meanwhile, the results showed that the oxidative induction time (OIT) of the polyethylene physically blended with antioxidant 4,4′-thiobis (2-tert-butyl-5-methylphenol) (antioxidant 300) and frequently used in cable insulation was 98.4 min, and the OIT value of polyethylene without any antioxidant was only 0.5 min [16].

Ultraviolet (UV) absorbers, such as 2-hydroxy-benzophenone, have been shown to be capable of absorbing harmful sunlight. The energy absorbed by the absorber is then dissipated through the formation of a reversible six-membered hydrogen bond ring system [15]. This process has been demonstrated to prevent polyethylene from undergoing photo-degradation by UV, thus acting as an antioxidant. Additionally, the absorbers have been found to act as polymer chain-breaking donors [14,15,16,17]. The anti-oxidation ability of 2-hydroxy-benzophenone was enhanced by the introduction of electron-donating substituent groups to the *para*-position of the hydroxyl groups, instead of the *ortho*-position [10,17]. The grafting of a UV absorber (4-allyloxy-2-hydroxyl-benzophenone) onto polyethylene has been utilized as a method of enhancing the electrical tree initiation voltage, thereby inhibiting its propagation and augmenting its breakdown strength properties [18].

This work sets out a new graftable antioxidant, which introduces electron-donating allyloxy groups on *para*-position to the hydroxyl groups, thus facilitating oxidation. In addition, the introduction of tertiary butyl groups serves to enhance the production of semi-quinones during the oxidation process. Evidently, it would be highly desirable for the new antioxidant to be grafted to polyethylene, thereby providing long-term protection against oxidative aging and, concomitantly, serving as a voltage stabilizer to resist high voltage. The new antioxidant with dual-functional use in a polyethylene system would further decrease the chemical impurities of the insulation materials.

To the best of our knowledge, no previous theoretical investigation has been performed on the anti-oxidative behavior of antioxidants (5ATB) at molecular and atom levels. We aim to design a new antioxidant and investigate the anti-oxidative reaction mechanisms and the grafting to polyethylene. The elucidation of the reaction mechanism is conducive to the design of highly efficient antioxidant molecules.

## 2. Computational Methods

In this work, the geometry structure optimization and frequency calculation of the stationary points for the studied six reaction channels on the ground state S_0_ (or the triplet state T_1_) were carried out by density functional theory (DFT) [18] at the B3LYP/6-311+G(*d,p*) level [19,20,21,22]. The B3LYP method was chosen in our present research based on the calculated values of the vibrational frequencies of 4-methylheptane and benzene at the B3LYP/6-311+G(*d,p*) level being in good agreement with the corresponding experimental data. The time-dependent density functional theory (TDDFT) method [23,24] was employed to calculate the excitation energies of the designed antioxidant molecules on the basis of the optimized geometries at the same level. The potential energy surface information was obtained along the minimum energy path (MEP) by the intrinsic reaction coordinate (IRC) theory with a gradient step-size of 0.05 (amu)^1/2^ Bohr [25]. Then, the first and second energy derivatives were obtained to calculate the curvature and the generalized vibrational frequencies along the reaction path. The values of *E*_g_, IP(*a*), and EA(*a*) were obtained based on the calculation results of the electronic structure. *E*_g_ refers to the energy gaps between the highest occupied molecular orbital (HOMO) and the lowest unoccupied molecular orbital (LUMO). IP(*a*) and EA(*a*) refer to the adiabatic ionization potentials and electron affinity energies, respectively. The natural charge population was analyzed by the natural bond orbital (NBO) method [26] based on the optimized geometries. All the electronic structure calculations of the stationary points were completed by the GAUSSIAN09 program package [27].

## 3. Results and Discussion

Aromatic carbonyl compounds characteristically undergo single electron excitation. The initial excited states are triplets [28,29], and a minimum energy crossing point has been theoretically identified [30]. In this work, the investigation of oxidative behavior during the UV radiation cross-linking process has been completed on the lowest triplet state. The molecular formulae, molecular names, and corresponding abbreviations of the studied molecules are listed in Table 1. 4-methylheptane (Pe) is selected as a model molecule of cross-linkable polyethylene. The newly designed antioxidant is named 5-allyloxy-2-hydroxyl-3-tert-butylbenzophenone (5ATB). In the course of the UV radiation cross-linking process, the ground state (S_0_) 5ATB is be excited to the singlet excited state S_1_ (n, π*) (with a calculated value of 3.14 eV) through the absorption of energy emitted from the optical system in the form of UV, and subsequently to the triplet excited state T_1_ (n, π*) (with a calculated value of 0.99 eV) by means of intersystem crossing (ISC).

Table 2 also lists the Gibbs free energy of the six reaction channels at the B3LYP/6-311+G (*d,p*) level at 298 K (ΔG^0^) and the reaction Gibbs energy barrier heights (ΔG^≠^) of the transition states (Supplementary Materials). Among them, the reaction channels ①, ①-1, and ①-2 are oxidative processes that occur during the preparation of polyethylene. It is widely acknowledged that polyethylene is susceptible to oxidation during its preparation (①). The free radicals of polyethylene and hydrogen peroxide, formed by the reaction of oxygen and polyethylene after hydrogen extraction, automatically combine (①-1). However, this peroxide is not stable, and the peroxide bond is prone to homolytic cleavage under high temperatures, resulting in the formation of oxide free radicals and hydroxyl free radicals (①-2). To prevent thermal oxidative aging, antioxidants must be introduced. Antioxidants 4AB and 5ATB react with O_2_ to exert antioxidant functions (② and ③). At the same time, antioxidant 5ATB also plays a repairing role in the cross-linking process, repairing excess free radicals in the cross-linking system to avoid affecting the cross-linking process of XLPE (④ to ⑥-1).

### 3.1. Stationary Point Geometries and NBO Charge Population

**Figure 1:**
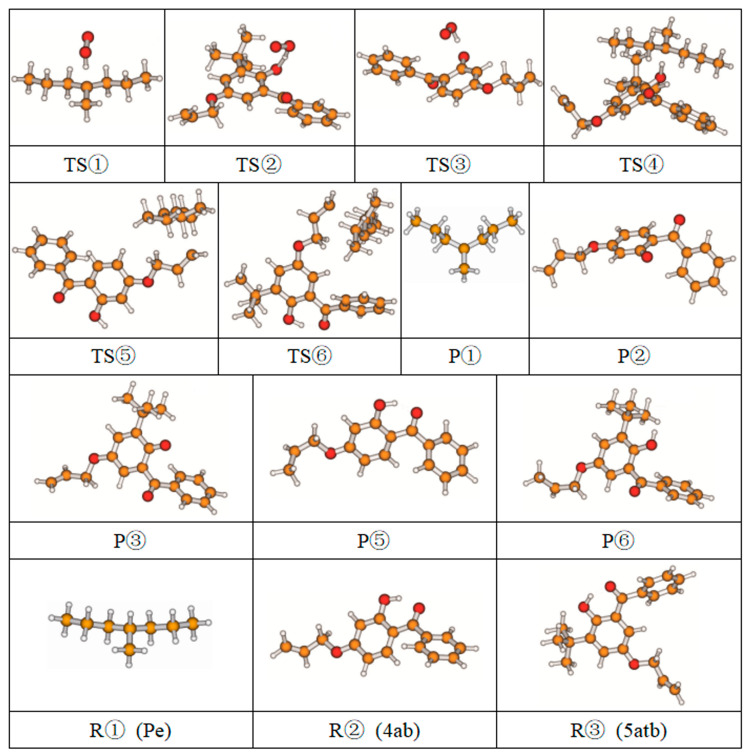
Optimized geometric structures of the six transition states at the B3LYP/6-311+G(*d,p*) level.

**Figure 2:**
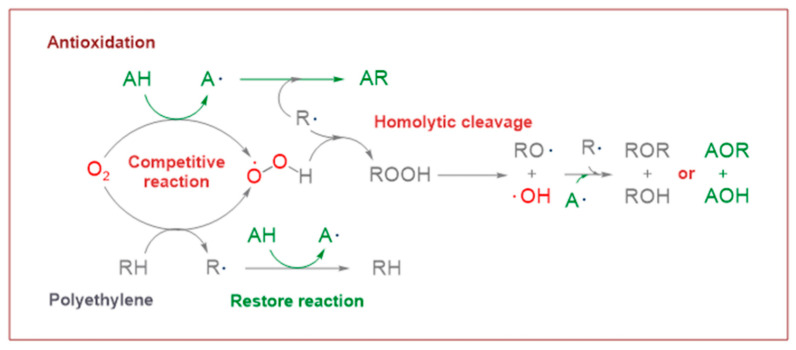
The overall reaction process of the newly designed antioxidant.

The optimized geometry structures of the stationary points of the six studied reaction channels, including the reactants, transition states, and products, are presented in Figure 1. The overall reaction process is illustrated in Figure 2, encompassing the oxidative reaction of polyethylene (ab. RH), the antioxidant behavior of the newly designed antioxidant (ab. AH), and the homolytic cleavage of the peroxide bond. The following table (see Table 2) presents a comprehensive listing of the bond lengths of the breaking and forming bonds (b/f) of the six transition states. Additionally, the corresponding bond lengths in equilibrium of the geometry structures of the reactants and products are also given. The labels of the reaction channels and transition states are consistent, with the reactants and products being abbreviated to the corresponding R and P, respectively.

**Table 2:** Optimized geometric structures (in angstrom) of transition states, reaction Gibbs energy barriers (ΔG^≠^), and reaction Gibbs free energies, ΔG^0^ (in eV).

	Reaction Equation	Reactant	b/f	Product	ΔG^≠^	ΔG^0^
①	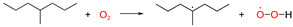	1.100	1.606/1.076	0.977	1.96	1.72
①-1	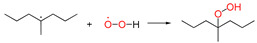	two radicals combined				
①-2	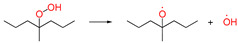	peroxide bond homolytic cleavage				
②	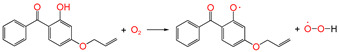	0.963	1.341/1.089	0.977	1.86	1.79
③	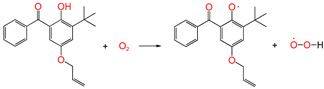	0.991	1.286/1.121	0.977	1.48	1.40
③-1	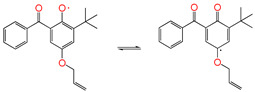	isomerization which is the same as keto-enol tautomerism				
④	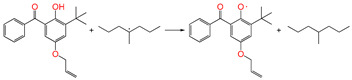	0.963	1.243/1.350	1.100	1.06	−3.31
④-1	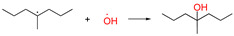	two radicals combined (it can also be Pe radicals or PeO radicals)				
④-2	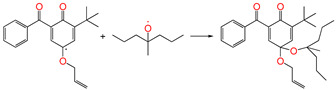	two radicals combined (it can also be OH radicals or Pe radicals)				
⑤	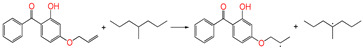	1.100	1.328/1.420	1.101	0.98	−0.96
⑥	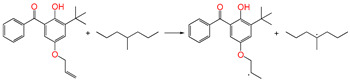	1.100	1.329/1.420	1.101	0.63	−0.46
⑥-1	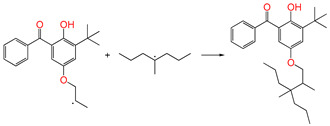	two radicals combined				

As illustrated in Table 2, the transition state structures of the H-abstraction reaction by O_2_ channels TS①, TS②, and TS③ exhibit a shared characteristic, namely, the elongation of the breaking bonds exceeds that of the forming bonds in their equilibrium molecules, respectively. This finding indicates that the H-abstraction reactions of Pe, 4AB, and 5ATB by O2 are all produce-like processes. It is evident that the aforementioned reaction channels will process via “later” transition states, which are consistent with Hammond’s postulate [31], as applied to endothermic reactions. The NBO calculation results for the six sites of the 4AB and 5ATB molecules are listed in Table 3. As illustrated in Table 3, the natural charge density on the H of the hydroxyl group (OH) in molecules 4AB and 5ATB is higher. This suggests that the covalent bonds between O and H are more susceptible to rupture, resulting in the formation of H radicals. The higher reactivity of the H of the hydroxyl group (OH) is the underlying reason for the antioxidant’s ability to function as an anti-oxidative. It has been established that the natural charge density on the C of CH_2_ in the C=C double bond of the 4AB and 5ATB molecules is higher than that of the C of CH in the C=C double bond. Furthermore, the negative charge on the C of CH_2_ in the C=C double bond is larger, and as a result, its capability of accepting H is also greater. The C of CH_2_ in the C=C double bond shows higher reactivity than the C of CH in the C=C double bond. It can be deduced that the grafting reaction of 4AB and 5ATB to the cross-linkable polyethylene molecule chain Pe would be initiated at the C site of CH_2_ in the C=C double bond.

### 3.2. Frontier MOs

The calculated values of adiabatic ionization potentials IP(*a*) and the adiabatic electron affinity energy EA(*a*), together with the relevant experimental data [32] (in brackets), are listed in Table 4, as well as the values of the HOMO-LUMO gap (*E_g_*).

For the newly designed antioxidant 5ATB (see Table 4), the *E*_g_ values of the aryl molecules (Bp, 4OB, 4AB, and 5ATB) are lower than those of the alkyl Pe molecule. It is evident that the introduction of a carbonyl group results in the formation of a π bond with the benzene ring. Conjugation effects occur between the benzene ring and the carbonyl group within the aryl molecules. In comparison with the σ bonds of Pe (*E*_g_ = 8.38 eV), it can be observed that the π bonds of aryl molecules possess higher HOMO and lower LUMO. This finding indicates that the *E*_g_ value of aryl molecules is relatively low, with 5ATB exhibiting the lowest *E*_g_ value of 3.66 eV.

For the newly designed antioxidant 5ATB, as illustrated in Table 4, the IP(*a*) value is found to be less than that of Pe, which is due to the *σ* electron of Pe being in the *sp*^n^ hybrid orbit, which is more strongly attracted to the atom’s nucleus and is not easy to dissociate. It has been established that, in comparison with the σ electron, the π electron of 5ATB is more readily dissociated due to its location in the p orbit, which is a greater distance from the nucleus. The IP(*a*) values of Bp, 4OB, 4AB, and 5ATB are all found to be less than Pe (9.41 eV), and the IP(*a*) value of 5ATB (7.18 eV) is the lowest of all.

For the newly designed antioxidant 5ATB in Table 4, the value of EA(*a*) is larger than Pe, as the heteroatom O is introduced in the aryl conjugate system. It is evident that the heteroatom O has the greater electronegativity, leading to a more potent binding force for the electron. There is the presence of large conjugated π bonds between the benzene ring and the carbonyl group in 5ATB, and tertiary butyl is the electron donating group. This results in an augmentation of the electron cloud density on the benzene ring, thereby facilitating electron acceptance. Furthermore, the distribution of the electronic cloud density of the 4AB molecule is not uniform, with a molecule density primarily at the 3- and 5-position, and the alkoxy is linked to the *meta*-position of the 2-hydroxyl groups. By contrast, the distribution of the electronic cloud density of the 5ATB molecule is uniform, with a molecule density primarily at the 4- and 6-position, and the tertiary butyl and alkoxy being linked to the *ortho*- and *para*-position of the 2-hydroxyl groups, respectively.

It is reported that polycyclic aromatic compounds and benzil-like structures are well suited to the role of voltage stabilizers [33,34]. It has been documented that the ultraviolet absorber (4AB) can be grafted onto polyethylene in order to serve as a voltage stabilizer, thereby enhancing its breakdown strength [17]. It is reported that voltage stabilizers have the capacity to capture high-energy electrons (termed “hot electrons”). These stabilizers then dissipate the energy of these electrons through impact, releasing non-harmful electrons of a lower energy. This process is intended to prevent the degradation of the polymer matrix. The EA(*a*) value of 5ATB (1.05 eV) is the largest. The results of this study indicate that the designed antioxidant 5ATB has the potential to serve as a voltage stabilizer, thereby providing a high voltage resistance performance.

### 3.3. Energetics

The reaction Gibbs free energies (ΔG^0^) and the reaction Gibbs energy barriers (ΔG^≠^) for the six reaction channels are also listed in Table 2. As demonstrated in Figure 2, the reaction process encompasses polyethylene cross-linking, oxidative reactions, anti-oxidative behavior, and the grafting reaction to polyethylene by UV radiation previously mentioned. From Table 2, it can be seen that the reaction process mainly includes four types of reactions, namely the oxidative reaction, the restore reaction of Pe radicals, the isomerization reaction which is the same as keto-enol tautomerism, and the grafting reaction.

#### 3.3.1. Oxidative Reaction

In the course of the research, it was established that the oxidative reaction of the newly designed antioxidant 5ATB (reaction channel ③) is easier than that of Pe (reaction channel ①). This is evidenced by the data in Table 2, which shows that the reaction Gibbs energy barrier ΔG^≠^_TS③_ (1.48 eV) is less than that of ΔG^≠^_TS①_ (1.96 eV). The newly designed antioxidant 5ATB can undergo a reaction with O_2_ forming relatively stable radicals before the polyethylene chain oxidation, which prevents the oxidation of the polyethylene chain. The lower reaction Gibbs energy barrier of the antioxidant is the reason why the antioxidant can play an anti-oxidative role. This is why the OIT value of antioxidant 300, which is frequently used in cable insulation, is 196 times higher than that without any antioxidants [15]. This is consistent with the analytic results of the natural charge density above. The calculated breaking bond dissociation energies ($D_{298}^{o}$) are 3.55 and 3.91 eV for 5ATB and Pe, respectively, at the B3LYP/6-311+G(*d,p*) level. The dissociation energy of the bonds is closely related to the corresponding reaction Gibbs energy barrier heights. The bond dissociation energy of the H atom in the hydroxyl group of the novel antioxidant is lower than that of Pe by approximately 0.36 eV. This means that the reactivity of the H of the hydroxyl groups on 5ATB is comparatively higher than that of Pe. The two radicals of the oxidative products combine to form peroxides (①-1 channel), and the peroxide bond homolytic cleavage to form the other two radicals (①-2 channel). Antioxidants are frequently employed to eliminate free radicals in XLPE insulation materials for high voltage cables. The reaction Gibbs energy barrier ΔG^≠^_TS③_ of 1.48 eV is lower than ΔG^≠^_TS②_ of 1.86 eV. This finding is consistent with the report that the anti-oxidation ability of 2-hydroxy-benzophenone was improved by the introduction of electron-donating substituent groups to the *para*-position of the hydroxyl groups [17].

#### 3.3.2. Restore Reaction

For the reaction channel ④, the aryl 5ATB possess a stronger *π*-electron delocalization ability than that of alkyl Pe, and the aromatic molecules are chosen as additives added in the XLPE material system, which can restore alkyl radicals through transforming the alkyl radicals to relatively stable aryl radicals. The restore reaction Gibbs energy barrier is 1.06 eV, which can prevent the formation of excessive amounts of polyethylene radicals in the cross-linking system, decrease the cross-linking density, and prevent the breakdown of polyethylene chains during service (the average carbon–carbon single bond energy is 3.60 eV). The two radicals, Pe and OH, combine to form the inactive product alcohol (reaction channel ④-1).

#### 3.3.3. Isomerization Reaction

The reaction channel ③-1 is an isomerization reaction that is analogous to the keto-enol tautomerism, wherein phenolic hydroxyl radicals are transferred to quinone-like structures. This reaction is reversible. The relatively stable 5ATB radicals can combine with the product of the ①-2 channel, forming the inactive product ether (④-2 channel), and can also be combined by OH radicals or Pe radicals to form inactive products.

#### 3.3.4. Grafting Reaction

Compared with the reaction channel ③, we can find from the calculated results in Table 2 that the reaction Gibbs energy barrier ΔG^≠^_TS⑥_ (0.63 eV) is lower than ΔG^≠^_TS③_ (1.48 eV), and that the reaction channel ⑥ is more advantageous in kinetics. This means that the grafting reaction ⑥ occurs before the oxidative reaction ③. In accordance with the analysis of natural charge density in Table 3, the grafting reaction of 5ATB to Pe can be initiated at the C site of CH_2_ in the C=C double bond. A similar case is also seen for the reaction channels ⑤ and ②. The grafting reaction of 4AB to the cross-linkable polyethylene molecule chain Pe can be initiated at the same C site of CH_2_ in the C=C double bond. The grafted antioxidants would prevent their own migration and provide long-term protection against oxidative aging resistance.

## 4. Conclusions

The theoretical calculation results of density functional theory were used to propose the antioxidant mechanism and grafting behavior of the newly designed antioxidant 5ATB after photocatalysis. This is the first time that these mechanisms have been proposed. The reaction potential energy information of the six reaction channels are obtained at the B3LYP/6-311+G(*d,p*) level. The analysis of frontier MOs, NBO charge populations, and the reaction potential energy information also confirmed that the designed 5ATB has dual functions of antioxidation and stabilizing voltage. The underlying mechanism of the designed antioxidant’s anti-oxidative effect was elucidated. The Gibbs energy barrier of the reactive antioxidant 5ATB with O_2_ was found to be approximately 0.48 eV lower than that of the polyethylene chain with O_2_. Additionally, the antioxidant 5ATB exhibited a more pronounced capacity to restore the polyethylene chains. The accepting H reaction of the C-site of CH₂ in the C=C double bond exhibits elevated reactivity, with a lower reaction Gibbs energy barrier height of 0.63 eV, as observed in the grafting of the antioxidant 5ATB onto polyethylene. The use of dual-functional antioxidants has been proven to reduce the introduction of chemical impurities and the electronic conductivity of insulating materials. The designed antioxidant 5ATB has the potential to exhibit excellent electrical performance and meet the requirements of insulation materials for future advanced power cables.

## Figures and Tables

**Table 1:** The molecular names, the molecular formulae, and the corresponding abbreviations (ab.) of the studied molecules.

ab.	Molecular Formula	Molecular Name
Pe	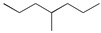	4-methylheptane (model molecule of polyethylene)
4OB	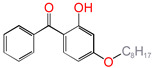	2-hydroxyl-4-*n*-octoxybenzophenone (ultraviolet absorber UV-531)
4AB	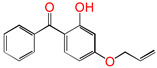	4-allyloxy-2-hydroxylbenzophenone (graftable UV absorber as voltage stabilizer)
5ATB	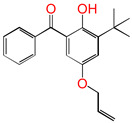	5-allyloxy-2-hydroxyl-3-tert-butylbenzophenone (designed as a new graftable antioxidant)

**Table 3:** Natural charge population on the T_1_ state of 4AB and 5ATB molecules.

States	Molecular Formula	Natural Charge Population			
		H of OH	O of OH	C of CH on C=C	C of CH_2_ on C=C
T_1_	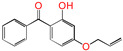	0.472	−0.638	−0.196	−0.348
	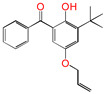	0.479	−0.606	−0.197	−0.348
S_0_	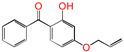	0.504	−0.671	−0.193	−0.353
	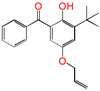	0.503	−0.686	−0.189	−0.359

**Table 4:** The calculated *E*_g_, IP(*a*), and EA(*a*) of the studied molecules, as well as the corresponding experimental data in brackets (in eV).

ab.	Molecular Formula	*E*_g_	IP(*a*)	EA(*a*)
Pe	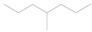	8.38	9.41	−1.09
Bp	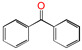	4.90	8.64(9.05)	0.73(0.69 ± 0.05)
4OB	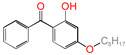	4.32	7.76	0.85
4AB	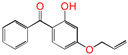	4.34	7.82	0.88
5ATB	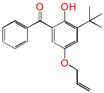	3.66	7.18	1.05

## Data Availability

The original contributions presented in this study are included in the article/Supplementary Materials. Further inquiries can be directed to the corresponding authors.

## Supplementary Materials

The following supporting information can be downloaded at: https://www.mdpi.com/article/10.3390/polym17040546/s1, Text: The optimized standard orientations of stationary points (reactants, products and transition states) of the six reaction channels at the B3LYP/6-311+G(*d*,*p*) level.
