# Supplementary material for: The Structural Design of a New Graftable Antioxidant and the Theoretical Study of Its Role in the Cross-Linking Reaction Process of Polyethylene
Source: Polymers (Basel). 2025 Feb 19;17(4):546. doi: 10.3390/polym17040546 (PMC11859502; doi:10.3390/polym17040546)
Supplement: Supplementary file 1 [file polymers-17-00546-s001.zip › polymers-3418724-supplementary.pdf]

Supplementary Materials

Structural Design of a New Graftable Antioxidant and Theoretical Study of the Role in the Cross-linking Reaction Process of Polyethylene

Yang Du [a], Hui Zhang [a,*], Chi Deng [a], Xia Du [a], Yan Shang [a], Xuan Wang [a,*], Qingguo Chen [a] and Zesheng Li [b]

[a] Key Laboratory of Engineering Dielectrics and Its Application of Ministry of Education & School of Material Science and Chemical Engineering, Harbin University of Science and Technology, Harbin 150080, P.R. China
[b] Key Laboratory of Cluster Science of Ministry of Education & School of Chemistry, Beijing Institute of Technology, Beijing 100081, P.R. China
[*] Correspondence: huizhang@hrbust.edu.cn; wangxuan@hrbust.edu.cn;

The optimized standard orientations of stationary points (reactants, products and transition states) of the six reaction channels at the B3LYP/6-311+G(*d,p*) level

TS①

6	0	-3.815119	-0.338294	-0.794146
6	0	-2.611830	-0.314108	0.153234
6	0	-1.294410	-0.623488	-0.574119
1	0	-1.185367	0.061515	-1.423369
6	0	-0.020322	-0.583945	0.246031
6	0	1.248668	-0.616167	-0.582112
8	0	0.014932	2.017120	0.892455
8	0	0.145060	2.657215	-0.240075
1	0	-0.004190	0.964919	0.669955
6	0	-0.018237	-1.294918	1.574786
1	0	-2.775836	-1.039915	0.955910
1	0	-2.542968	0.668739	0.631861
1	0	-3.706818	0.404408	-1.590372
1	0	-3.927047	-1.318777	-1.267852
1	0	-4.743438	-0.119475	-0.260058
1	0	1.170656	0.146549	-1.366672
6	0	2.580611	-0.438808	0.160868
1	0	1.273078	-1.580730	-1.121404
1	0	-1.371277	-1.630736	-1.022706
1	0	0.865219	-1.057510	2.170069
1	0	-0.901119	-1.059255	2.171773
1	0	-0.017462	-2.386707	1.419475
6	0	3.774429	-0.383530	-0.797036

1	0	2.548864	0.480970	0.755277
1	0	2.727061	-1.260844	0.868864
1	0	4.714117	-0.264446	-0.251352
1	0	3.848948	-1.300132	-1.390891
1	0	3.684897	0.456293	-1.492582

TS②

6	0	-0.040433	-1.680743	0.014815
6	0	-1.316018	-1.969893	-0.457871
6	0	-2.348636	-0.995585	-0.403624
6	0	-2.087402	0.250610	0.118913
6	0	-0.780967	0.575699	0.608778
6	0	0.256666	-0.438881	0.561768
1	0	-1.551226	-2.940303	-0.877658
1	0	-2.835292	1.026133	0.189378
8	0	-0.540426	1.744610	1.070118
8	0	0.330022	2.984601	-0.804382
8	0	0.358688	2.014235	-1.658847
1	0	-0.036217	2.550222	0.124370
8	0	-3.530593	-1.412786	-0.921202
6	0	-4.654316	-0.513059	-0.969902
1	0	-5.321354	-0.986347	-1.694171
6	0	-5.346584	-0.370858	0.355207
1	0	-4.346765	0.456355	-1.374813
6	0	-5.758699	0.786375	0.863852
1	0	-5.541256	-1.303042	0.879968
1	0	-6.301382	0.832275	1.800714
1	0	0.726246	-2.445841	-0.031432
6	0	1.582011	-0.201173	1.241282
8	0	1.587781	0.003322	2.440255
6	0	2.840870	-0.287816	0.444932
6	0	2.841424	-0.236061	-0.954985
6	0	4.043767	-0.292497	-1.656239
6	0	5.248664	-0.409340	-0.966356
6	0	5.255126	-0.459141	0.430062
6	0	4.059460	-0.392572	1.132660
1	0	1.908853	-0.122946	-1.493282
1	0	4.039317	-0.238626	-2.738750
1	0	6.183516	-0.456638	-1.513818
1	0	6.193762	-0.546695	0.965309
1	0	4.043233	-0.418449	2.215460
1	0	-5.574812	1.731667	0.361444

TS③

6	0	3.651298	-2.108275	0.449827
6	0	2.540594	-1.476430	-0.125961
6	0	2.661761	-0.898891	-1.395483
6	0	3.873089	-0.952884	-2.078386
6	0	4.978298	-1.566900	-1.490767
6	0	4.866192	-2.142017	-0.223638
6	0	1.241011	-1.528762	0.607603
8	0	1.041989	-2.369144	1.468244
6	0	0.109320	-0.598148	0.234129
6	0	-1.135467	-1.177134	0.067598
6	0	-2.222344	-0.371623	-0.290770
6	0	-2.052153	1.019825	-0.473542
6	0	-0.844488	1.651041	-0.279913
6	0	0.290136	0.827029	0.116018
8	0	-3.475798	-0.823724	-0.491684
6	0	-3.762927	-2.228105	-0.317928
6	0	-5.211025	-2.440696	-0.624116
6	0	-6.060005	-3.036525	0.207305
6	0	-0.686927	3.167039	-0.484203
6	0	-2.004445	3.819314	-0.947113
8	0	1.447889	1.346133	0.353610
6	0	-0.269464	3.848121	0.842494
6	0	0.381165	3.437663	-1.573026
8	0	1.709624	1.318308	2.729773
8	0	0.534649	0.895531	3.052294
1	0	1.724447	1.345591	1.609701
1	0	-2.935031	1.571491	-0.764031
1	0	-1.237919	-2.241900	0.223657
1	0	1.809120	-0.414983	-1.855467
1	0	3.956842	-0.511720	-3.064997
1	0	5.924869	-1.598375	-2.018866
1	0	5.725531	-2.618424	0.234383
1	0	3.541556	-2.563498	1.426384
1	0	-3.132625	-2.800391	-1.009490
1	0	-3.526542	-2.530020	0.706785
1	0	-5.542585	-2.096219	-1.599991
1	0	-7.094820	-3.205844	-0.066352
1	0	-5.750823	-3.381151	1.189215
1	0	-0.244268	4.932717	0.700611
1	0	-0.986472	3.628670	1.638072
1	0	0.717418	3.528425	1.170092
1	0	0.086162	2.988792	-2.526553
1	0	0.479524	4.516120	-1.728490
1	0	1.353666	3.041151	-1.285557

1	0	-1.837949	4.889050	-1.094647
1	0	-2.357544	3.409185	-1.897634
1	0	-2.801087	3.712652	-0.205493

TS④

6	0	-1.332139	-1.614742	1.196708
6	0	-0.243948	-0.971028	0.605057
6	0	-0.432532	-0.051668	-0.481329
6	0	-1.723232	0.034926	-1.083922
6	0	-2.774981	-0.668424	-0.490680
6	0	-2.600508	-1.463914	0.652628
6	0	1.114933	-1.275961	1.135037
8	0	1.145585	-0.924369	2.399281
8	0	0.591214	0.636834	-0.971850
6	0	-1.961822	0.870575	-2.360510
6	0	-1.696279	2.368259	-2.086475
8	0	-3.596611	-2.162395	1.272287
6	0	-4.942055	-2.116415	0.780183
6	0	-5.664249	-0.853984	1.159898
6	0	-6.474518	-0.177932	0.350604
6	0	2.180647	-1.939769	0.472564
6	0	3.384858	-2.232976	1.172907
6	0	4.416060	-2.915573	0.548470
6	0	4.300885	-3.325744	-0.784517
6	0	3.121185	-3.042841	-1.485350
6	0	2.076062	-2.367483	-0.877704
6	0	-1.028065	0.369665	-3.489340
6	0	-3.411188	0.746203	-2.872824
6	0	1.813909	2.414618	0.449792
6	0	1.183577	2.499856	1.834124
6	0	-0.288962	2.926421	1.918957
6	0	-0.813885	2.898675	3.358083
6	0	1.626488	3.632300	-0.435628
6	0	3.233125	1.855829	0.483598
6	0	3.905050	1.566538	-0.865947
6	0	5.315342	0.992867	-0.697911
1	0	-1.185881	-2.270355	2.044715
1	0	1.939828	3.445173	-1.464054
1	0	1.306432	1.535894	2.337644
1	0	-3.763148	-0.574294	-0.909719
1	0	1.788494	3.214808	2.419083
1	0	3.239024	0.944307	1.089496
1	0	3.850913	2.585263	1.036438
1	0	0.592448	3.977133	-0.454541

1	0	2.241889	4.460893	-0.052888
1	0	1.145259	1.439605	-0.201790
1	0	-1.896593	2.950114	-2.991725
1	0	-2.352264	2.742800	-1.295129
1	0	-0.663592	2.540639	-1.794240
1	0	-3.514763	1.336234	-3.786919
1	0	-3.674199	-0.286500	-3.119133
1	0	-4.140382	1.130941	-2.154179
1	0	-1.207779	0.947311	-4.401460
1	0	0.020646	0.475196	-3.215341
1	0	-1.224122	-0.682707	-3.715842
1	0	-0.416878	3.934429	1.511195
1	0	-0.900094	2.262530	1.298418
1	0	3.287775	0.861433	-1.430652
1	0	3.962238	2.483784	-1.461443
1	0	5.783023	0.807664	-1.668798
1	0	5.961150	1.682665	-0.144001
1	0	5.291936	0.043984	-0.155507
1	0	-1.862224	3.206657	3.403810
1	0	-0.740944	1.894295	3.785143
1	0	-0.241186	3.574738	4.001260
1	0	-5.416668	-2.973496	1.265021
1	0	-4.969554	-2.288752	-0.301329
1	0	-5.519800	-0.531705	2.188361
1	0	-7.015247	0.697422	0.691249
1	0	-6.635718	-0.479108	-0.680633
1	0	1.169599	-2.164761	-1.434535
1	0	3.019626	-3.357921	-2.518508
1	0	5.111614	-3.858442	-1.267310
1	0	5.322165	-3.134370	1.103864
1	0	3.482915	-1.923732	2.207378

TS⑤

6	0	-5.993845	-1.092916	-1.041241
6	0	-4.877221	-0.676448	-0.302790
6	0	-4.273993	-1.575829	0.585322
6	0	-4.775738	-2.868218	0.728697
6	0	-5.875703	-3.277422	-0.022357
6	0	-6.483963	-2.386391	-0.909132
6	0	-4.419477	0.748868	-0.452840
8	0	-5.177787	1.591171	-0.898228
6	0	-3.012542	1.098917	-0.071011
6	0	-2.712710	2.271361	0.656993
6	0	-1.392859	2.622809	0.914190

6	0	-0.338109	1.835111	0.440976
6	0	-0.611139	0.671679	-0.285600
6	0	-1.940072	0.323528	-0.516110
8	0	0.906029	2.285665	0.747765
6	0	2.044835	1.534306	0.315377
6	0	3.270506	2.244121	0.769500
6	0	4.624741	1.675614	0.646730
8	0	-3.746393	3.014496	1.134087
6	0	4.853200	-0.921000	-0.198927
6	0	4.107188	-1.818483	0.788875
6	0	4.454145	-1.662334	2.276244
6	0	3.607702	-2.572214	3.172384
6	0	6.367060	-1.063761	-0.148794
6	0	4.257958	-1.022820	-1.603566
6	0	4.811531	-0.067771	-2.670303
6	0	4.100249	-0.221916	-4.018583
1	0	4.204930	-1.239896	-4.407040
1	0	3.029666	-0.011555	-3.930016
1	0	4.511277	0.462096	-4.765961
1	0	5.882713	-0.243601	-2.810442
1	0	4.712875	0.966941	-2.323703
1	0	3.171659	-0.880693	-1.535689
1	0	4.392635	-2.058266	-1.958092
1	0	4.282018	-2.867336	0.496774
1	0	3.028151	-1.661047	0.663682
1	0	4.309829	-0.619232	2.577894
1	0	5.512956	-1.886283	2.439697
1	0	3.753305	-3.626519	2.916523
1	0	3.869637	-2.447552	4.226558
1	0	2.540738	-2.351478	3.067328
1	0	6.756563	-0.918560	0.861131
1	0	6.671235	-2.067857	-0.477105
1	0	6.867711	-0.342401	-0.798259
1	0	-4.309938	-3.553174	1.428302
1	0	-3.425463	-1.259610	1.179322
1	0	-6.463654	-0.382606	-1.710711
1	0	-7.342131	-2.702109	-1.491816
1	0	-6.261989	-4.284982	0.085437
1	0	-2.149020	-0.575338	-1.084604
1	0	-1.157039	3.512929	1.488212
1	0	0.180348	0.044565	-0.669887
1	0	-3.403565	3.772491	1.619071
1	0	2.018198	1.436237	-0.783998
1	0	1.997080	0.515375	0.727159

1	0	3.136727	3.275079	1.088849
1	0	4.639498	0.327623	0.199422
1	0	5.264867	2.157260	-0.100983
1	0	5.172908	1.586095	1.589751

TS⑥

6	0	0.142812	2.558785	-1.173925
6	0	-0.643315	2.924528	-0.074255
6	0	-0.212035	3.965257	0.758702
6	0	1.000819	4.601894	0.519006
6	0	1.780084	4.231558	-0.577490
6	0	1.343397	3.218301	-1.428827
6	0	-1.994514	2.328269	0.182751
8	0	-2.913884	3.111179	0.472516
6	0	-2.229910	0.878965	0.090669
6	0	-1.150549	-0.038343	0.059550
6	0	-1.407041	-1.390424	-0.025586
6	0	-2.736770	-1.842190	-0.100294
6	0	-3.831771	-0.994181	-0.055504
6	0	-3.564681	0.397549	0.072780
8	0	-0.462534	-2.381088	-0.044585
6	0	0.911502	-2.024855	0.084676
6	0	1.715428	-3.277610	0.084380
6	0	3.186761	-3.306066	0.161669
8	0	-4.603531	1.247069	0.171719
6	0	-5.274230	-1.537808	-0.119535
6	0	-6.015085	-0.931210	-1.336037
6	0	-5.299466	-3.070841	-0.280329
6	0	-6.030351	-1.195038	1.187571
6	0	4.540561	-0.926875	0.172648
6	0	4.058814	-0.096264	1.362378
6	0	4.095289	-0.765803	2.743323
6	0	3.543432	0.141708	3.847669
6	0	5.958447	-1.459638	0.316357
6	0	4.271178	-0.212640	-1.152004
6	0	4.543116	-1.001594	-2.440417
6	0	4.195249	-0.202315	-3.700484
1	0	6.230903	-2.123244	-0.507294
1	0	6.091646	-2.020548	1.243879
1	0	6.683409	-0.633067	0.325308
1	0	3.227858	0.127677	-1.161935
1	0	4.876905	0.708772	-1.171782
1	0	3.964398	-1.931526	-2.429943
1	0	5.596660	-1.295015	-2.485472

1	0	4.402313	-0.777483	-4.606985
1	0	3.135254	0.070894	-3.717315
1	0	4.776993	0.723440	-3.754943
1	0	3.034337	0.242697	1.163554
1	0	4.664561	0.824225	1.404371
1	0	5.121885	-1.049279	2.996000
1	0	3.517892	-1.696497	2.715348
1	0	3.578824	-0.351720	4.822765
1	0	2.502336	0.417401	3.652688
1	0	4.120969	1.068493	3.923130
1	0	3.790663	-2.022462	0.159590
1	0	3.684493	-3.771400	-0.695338
1	0	3.593314	-3.726269	1.088256
1	0	1.150138	-4.206390	0.088219
1	0	1.206670	-1.357780	-0.742975
1	0	1.058427	-1.451267	1.016924
1	0	-2.872490	-2.911716	-0.178846
1	0	-0.141596	0.337458	0.129855
1	0	-4.225123	2.145771	0.346526
1	0	-0.839373	4.267706	1.588413
1	0	1.333535	5.394744	1.179281
1	0	2.719334	4.737011	-0.772173
1	0	1.934887	2.943110	-2.294590
1	0	-0.198175	1.782090	-1.847877
1	0	-7.030257	-1.336796	-1.388764
1	0	-6.083480	0.153213	-1.268554
1	0	-5.502504	-1.189133	-2.267659
1	0	-6.338690	-3.405235	-0.332634
1	0	-4.803220	-3.397922	-1.198480
1	0	-4.833923	-3.583245	0.566229
1	0	-7.044150	-1.605055	1.143969
1	0	-5.526715	-1.637628	2.052091
1	0	-6.102347	-0.120027	1.343307

R① (Pe)

6	0	0.703811	1.834928	0.000000
1	0	1.721300	2.236121	0.000000
6	0	0.703811	0.303946	0.000000
1	0	0.191412	2.230936	0.882787
1	0	0.191412	2.230936	-0.882787
6	0	-0.703811	-0.303946	0.000000
1	0	1.253169	-0.061796	-0.876297
1	0	1.253169	-0.061796	0.876297
6	0	-0.703811	-1.834928	0.000000

1	0	-1.253169	0.061796	0.876297
1	0	-1.253169	0.061796	-0.876297
1	0	-1.721300	-2.236121	0.000000
1	0	-0.191412	-2.230936	-0.882787
1	0	-0.191412	-2.230936	0.882787

R② (4ab)

6	0	2.998132	0.076171	0.043574
6	0	4.138526	0.431684	-0.688325
6	0	5.267307	-0.380233	-0.670454
6	0	5.282796	-1.539044	0.106641
6	0	4.163601	-1.883316	0.862244
6	0	3.021512	-1.086065	0.824402
1	0	2.161080	-1.351500	1.426766
1	0	4.179450	-2.770983	1.484448
1	0	6.166880	-2.166286	0.129521
1	0	6.138704	-0.105547	-1.253976
1	0	4.126219	1.347972	-1.266011
6	0	1.830604	1.015415	0.033492
8	0	2.069506	2.233120	0.114142
6	0	0.454978	0.526601	-0.066365
6	0	-0.622057	1.433333	0.145491
6	0	-1.950189	0.992546	0.085317
6	0	-2.226433	-0.333460	-0.229363
6	0	-1.176886	-1.238300	-0.493842
6	0	0.125504	-0.805833	-0.409101
1	0	0.922701	-1.501553	-0.634250
1	0	-1.422058	-2.256363	-0.766878
1	0	-2.725406	1.721433	0.273541
8	0	-0.419389	2.728415	0.414030
1	0	0.557610	2.882552	0.344709
8	0	-3.474588	-0.850910	-0.320282
6	0	-4.606685	0.008454	-0.106080
1	0	-4.558724	0.841013	-0.819737
1	0	-4.581077	0.417838	0.909338
6	0	-5.846219	-0.799164	-0.328122
6	0	-6.841954	-0.884262	0.548569
1	0	-6.798131	-0.386600	1.512574
1	0	-7.739845	-1.451400	0.332382
1	0	-5.906861	-1.312741	-1.284006

R③ (5atb)

6	0	-1.340868	-1.218034	-0.102845
6	0	0.018689	-0.813307	-0.135768

6	0	0.342586	0.565087	-0.103115
6	0	-0.664243	1.500388	0.002457
6	0	-2.001529	1.076044	0.091175
6	0	-2.379957	-0.256569	0.043474
6	0	1.069541	-1.837378	-0.245036
8	0	0.793455	-3.013449	-0.531251
8	0	-0.486079	2.859576	0.024505
6	0	0.848624	3.368189	-0.026457
6	0	0.782739	4.858901	0.092288
6	0	1.366345	5.699551	-0.756357
8	0	-1.673420	-2.517976	-0.203802
6	0	2.513156	-1.506458	-0.011953
6	0	2.941195	-0.743258	1.081542
6	0	4.301169	-0.541184	1.307017
6	0	5.243096	-1.080447	0.432659
6	0	4.823303	-1.844838	-0.656462
6	0	3.467514	-2.071510	-0.867755
1	0	-2.744247	1.855798	0.185436
1	0	4.624300	0.033740	2.167488
1	0	2.214049	-0.333685	1.772315
1	0	3.133055	-2.687868	-1.693414
1	0	5.553732	-2.272311	-1.333932
1	0	6.300629	-0.913849	0.604079
1	0	1.376170	0.861349	-0.192009
6	0	-3.862514	-0.674750	0.127763
1	0	1.427063	2.946959	0.808314
1	0	1.335494	3.073195	-0.963962
1	0	0.230825	5.235668	0.949562
1	0	1.321445	6.772793	-0.611809
1	0	1.913029	5.345469	-1.625111
1	0	-0.837292	-3.016357	-0.387538
6	0	-4.081235	-1.609229	1.342440
6	0	-4.789114	0.543565	0.310783
6	0	-4.288192	-1.390687	-1.177109
1	0	-5.138074	-1.885773	1.408581
1	0	-3.493866	-2.522338	1.262158
1	0	-3.808831	-1.102261	2.272985
1	0	-5.823375	0.196376	0.377311
1	0	-4.569198	1.095312	1.229254
1	0	-4.732155	1.238371	-0.531942
1	0	-5.346135	-1.664723	-1.117861
1	0	-4.161116	-0.729243	-2.039259
1	0	-3.708975	-2.296286	-1.348720

P①

6	0	-3.377148	-1.107769	-0.053194
6	0	-2.020424	-0.538516	-0.477733
6	0	-1.309032	0.231377	0.662710
6	0	0.000003	0.855517	0.280524
6	0	0.000002	2.177866	-0.422566
6	0	1.309096	0.231480	0.662686
6	0	2.020370	-0.538602	-0.477695
6	0	3.377127	-1.107792	-0.053223
1	0	3.855451	-1.652122	-0.872386
1	0	-1.163172	-0.458062	1.503632
1	0	-2.000906	1.010250	1.019061
1	0	-2.154121	0.130890	-1.334812
1	0	-1.369369	-1.349666	-0.821107
1	0	-3.855680	-1.651774	-0.872466
1	0	-3.270351	-1.800530	0.787746
1	0	-4.059605	-0.311407	0.260696
1	0	2.000975	1.010453	1.018805
1	0	1.163356	-0.457812	1.503747
1	0	1.369266	-1.349789	-0.820882
1	0	2.153966	0.130660	-1.334905
1	0	4.059696	-0.311358	0.260237
1	0	3.270464	-1.800240	0.787991
1	0	0.884019	2.310942	-1.054925
1	0	-0.884484	2.311336	-1.054167
1	0	0.000523	3.016864	0.294678

P②

6	0	2.644252	-1.058288	-0.640061
6	0	2.952833	0.174272	-0.052619
6	0	4.277004	0.445948	0.318707
6	0	5.270056	-0.504698	0.119750
6	0	4.954994	-1.730520	-0.470199
6	0	3.643434	-2.002993	-0.855174
6	0	1.934679	1.247050	0.130124
8	0	2.261680	2.408756	0.316200
6	0	0.464462	0.940564	0.029202
6	0	-0.328866	1.756080	-0.764258
6	0	-1.700791	1.546229	-0.873010
6	0	-2.340277	0.505336	-0.138958
6	0	-1.601706	-0.300593	0.690812
6	0	-0.168334	-0.115443	0.826882
8	0	0.492421	-0.814071	1.621823
8	0	-3.678349	0.423639	-0.340785

6	0	-4.420264	-0.572645	0.384301
6	0	-5.865209	-0.424353	0.027914
6	0	-6.622703	-1.421837	-0.417590
1	0	1.627349	-1.280517	-0.937705
1	0	3.398135	-2.952674	-1.316489
1	0	5.730994	-2.471173	-0.628866
1	0	6.289704	-0.293647	0.421409
1	0	4.504236	1.406833	0.763544
1	0	0.133586	2.567843	-1.313339
1	0	-2.314061	2.170658	-1.511287
1	0	-2.035389	-1.085147	1.295057
1	0	-4.268355	-0.409348	1.459143
1	0	-4.051088	-1.571790	0.128888
1	0	-6.218704	-2.416197	-0.581602
1	0	-7.676156	-1.281647	-0.629812
1	0	-6.287639	0.565586	0.178207

P③

6	0	0.974488	-1.126555	-0.216745
6	0	-0.236285	-0.516173	-0.466611
6	0	-0.373460	0.939189	-0.464737
6	0	0.812365	1.739576	-0.092386
6	0	1.978350	1.073367	0.186933
6	0	2.085075	-0.338992	0.123007
6	0	-1.393365	-1.402294	-0.854989
8	0	-1.204906	-2.267894	-1.695097
8	0	-1.453362	1.463747	-0.806431
6	0	0.717252	3.269873	-0.050568
6	0	2.044197	3.915101	0.391675
8	0	3.308669	-0.827778	0.407981
6	0	3.537573	-2.252331	0.360318
6	0	4.959421	-2.501166	0.750869
6	0	5.816074	-3.196702	0.009589
6	0	-2.712475	-1.268889	-0.170412
6	0	-3.828842	-1.883934	-0.752826
6	0	-5.067069	-1.829818	-0.124692
6	0	-5.199617	-1.178204	1.102860
6	0	-4.090609	-0.577067	1.696320
6	0	-2.854190	-0.614411	1.058637
6	0	0.372737	3.809307	-1.461978
6	0	-0.379560	3.699421	0.955592
1	0	2.881524	1.603302	0.457875
1	0	-4.190700	-0.074052	2.651335
1	0	-1.998680	-0.139242	1.522257

1	0	-3.704655	-2.396058	-1.698972
1	0	-5.929634	-2.295519	-0.587825
1	0	-6.165146	-1.139467	1.595008
1	0	1.043057	-2.203450	-0.291881
1	0	2.852145	-2.739921	1.064115
1	0	3.333241	-2.626603	-0.647316
1	0	5.262834	-2.094135	1.711673
1	0	6.828892	-3.387664	0.344445
1	0	5.534652	-3.605078	-0.956161
1	0	-0.428770	4.791745	0.997992
1	0	-1.357330	3.320676	0.662200
1	0	-0.147825	3.336301	1.961806
1	0	0.328973	4.902401	-1.433408
1	0	1.142201	3.523452	-2.185382
1	0	-0.588153	3.431989	-1.807617
1	0	1.922418	5.000828	0.416587
1	0	2.344553	3.595841	1.393993
1	0	2.860831	3.694263	-0.301489

P⑤

6	0	2.982110	-1.166397	0.771563
6	0	3.010543	0.034448	0.051827
6	0	4.172385	0.385070	-0.647980
6	0	5.270860	-0.467551	-0.659820
6	0	5.234541	-1.664708	0.056132
6	0	4.094024	-2.006063	0.780485
6	0	1.877824	1.015496	0.078496
8	0	2.160805	2.217671	0.225921
6	0	0.487465	0.583826	-0.061220
6	0	0.113395	-0.715933	-0.477033
6	0	-1.202573	-1.094356	-0.598321
6	0	-2.223959	-0.166909	-0.297968
6	0	-1.901308	1.130645	0.086978
6	0	-0.559056	1.517513	0.185236
8	0	-0.312232	2.788978	0.522453
8	0	-3.487093	-0.632270	-0.431754
6	0	-4.604924	0.258643	-0.146174
6	0	-5.871491	-0.493616	-0.324698
6	0	-6.274754	-1.565053	0.625366
1	0	2.104971	-1.430373	1.349918
1	0	4.069900	-2.924377	1.356227
1	0	6.095180	-2.324161	0.056290
1	0	6.159194	-0.195248	-1.218416
1	0	4.200698	1.329901	-1.177071

1	0	0.887277	-1.428664	-0.729230
1	0	-1.480478	-2.087072	-0.927792
1	0	-2.650768	1.879333	0.299342
1	0	0.670612	2.909514	0.472537
1	0	-4.550920	1.118076	-0.820763
1	0	-4.498670	0.611266	0.887893
1	0	-6.043331	-1.293212	1.661444
1	0	-7.341982	-1.789080	0.554753
1	0	-6.410470	-0.378796	-1.258467
1	0	-5.733541	-2.502471	0.423168

P⑥

6	0	0.899858	-1.188263	-0.193852
6	0	-0.294322	-0.510051	-0.469951
6	0	-0.344007	0.886928	-0.426359
6	0	0.799952	1.642909	-0.069165
6	0	1.957679	0.928345	0.235272
6	0	2.026958	-0.467965	0.174644
6	0	-1.472701	-1.353952	-0.883605
8	0	-1.322080	-2.209359	-1.740240
8	0	-1.541290	1.447462	-0.802162
6	0	0.794194	3.189662	-0.028348
6	0	2.162070	3.758973	0.400034
8	0	3.241898	-1.012764	0.493634
6	0	3.389642	-2.451626	0.406972
6	0	4.791012	-2.811302	0.743672
6	0	5.920080	-2.432725	-0.148451
6	0	-2.789932	-1.202037	-0.188113
6	0	-3.934204	-1.728751	-0.800820
6	0	-5.168220	-1.654888	-0.165210
6	0	-5.268561	-1.073615	1.100128
6	0	-4.131341	-0.561380	1.723335
6	0	-2.898776	-0.617329	1.078425
6	0	0.499711	3.769466	-1.436750
6	0	-0.236053	3.708158	1.008874
1	0	2.864184	1.441893	0.521422
1	0	-4.205653	-0.118408	2.710198
1	0	-2.016015	-0.217842	1.563056
1	0	-3.834468	-2.191528	-1.775034
1	0	-6.051980	-2.053229	-0.650917
1	0	-6.230003	-1.022863	1.599306
1	0	0.903649	-2.265732	-0.278147
1	0	2.682202	-2.929002	1.093381
1	0	3.143425	-2.761324	-0.618644

1	0	5.001614	-3.198651	1.734326
1	0	6.813085	-3.030652	0.050747
1	0	5.652929	-2.546448	-1.205203
1	0	-1.518676	2.399066	-0.683394
1	0	-0.219867	4.801490	1.036562
1	0	-1.269825	3.409738	0.813104
1	0	0.013115	3.339900	2.007312
1	0	0.501696	4.862810	-1.398458
1	0	1.271458	3.453953	-2.143223
1	0	-0.458677	3.458734	-1.858923
1	0	2.106847	4.850549	0.416322
1	0	2.448058	3.428170	1.401438
1	0	2.956011	3.479904	-0.296481
1	0	6.200439	-1.376596	-0.012993
